# Control of Glucose, Blood Pressure, and Cholesterol among Adults with Diabetes: The Brazilian National Health Survey

**DOI:** 10.3390/jcm10153428

**Published:** 2021-07-31

**Authors:** Rodrigo Citton P. dos Reis, Bruce B. Duncan, Célia Landmann Szwarcwald, Deborah Carvalho Malta, Maria Inês Schmidt

**Affiliations:** 1 Postgraduate Program in Epidemiology, Universidade Federal do Rio Grande do Sul, Porto Alegre 90035-003, RS, Brazil; bbduncan@ufrgs.br (B.B.D.); maria.schmidt@ufrgs.br (M.I.S.); 2 Statistics Department, Universidade Federal do Rio Grande do Sul, Porto Alegre 91509-900, RS, Brazil; 3 Social Medicine Department, Universidade Federal do Rio Grande do Sul, Porto Alegre 90035-003, RS, Brazil; 4 Hospital de Clínicas de Porto Alegre, Porto Alegre 90035-003, RS, Brazil; 5 Institute of Communication and Scientific and Technological Information on Health of Oswaldo Cruz Foundation, Rio de Janeiro 21040-900, RJ, Brazil; celia.szwarcwald@icict.fiocruz.br; 6 Department of Maternal and Child Nursing and Public Health, School of Nursing, Universidade Federal de Minas Gerais, Belo Horizonte 30130-100, MG, Brazil; dcmalta@uol.com.br

**Keywords:** diabetes mellitus, glucose control, blood pressure, LDL cholesterol, measuring quality of diabetes care

## Abstract

ABC (glucose, blood pressure and LDL-cholesterol) goals are basic standards of diabetes care. We aimed to assess ABC control and related factors in a representative sample of Brazilian adults with diabetes. We analyzed 465 adults with known diabetes in the Brazilian National Health Survey. The targets used were <7% for glycated hemoglobin (A1C); <140/90 mmHg for blood pressure; and <100 mg/dL for LDL-C, with stricter targets for the latter two for those with high cardiovascular (CVD) risk. Individual goals were attained by 46% (95% CI, 40.3–51.6%) for A1C, 51.4% (95% CI, 45.7–57.1%) for blood pressure, and 40% (95% CI, 34.5–45.6%) for LDL-C. The achievement of all three goals was attained by 12.5% (95% CI, 8.9–16.2%). Those with high CVD risk attained blood pressure and LDL-C goals less frequently. A1C control improved with increasing age and worsened with greater duration of diabetes. Achievement of at least two ABC goals decreased with increasing BMI and greater duration of diabetes. In sum, about half of those with known diabetes achieved each ABC goal and only a small fraction achieved all three goals. Better access and adherence to treatment and strategies to personalize goals according to specific priorities are of the essence.

## 1. Introduction

Brazil, in 2019, ranked fifth among countries regarding the absolute number of adults with diabetes (16.8 million) [1]. Diabetes mellitus was Brazil’s fifth leading cause of disease burden in 2019 [2], and is expected to rise to the third position by 2040 [3]. The risk of long-term complications, particularly cardiovascular ones, is high for people with diabetes [4,5], imposing high costs on patients, families, health systems and nations.

Improved control of glucose, blood pressure and cholesterol levels of people diagnosed with diabetes reduces the risk of long-term complications [6,7,8,9,10]. Multifactorial intervention has been stimulated by the major protection found in a Danish randomized trial [6], although subsequent studies have questioned this result [11]. Control of major risk factors, known as the ABC control of diabetes, has become part of major international guidelines for diabetes.

The American Diabetes Association (ADA) “Standards of Medical Care in Diabetes” recommend goals for glycated hemoglobin A1c (A1C) of less than 7% and blood pressure less than 140/90 mmHg. Statin treatment is recommended for patients over 40 and to be considered in younger adults, although no low-density lipoprotein cholesterol (LDL-C) target is established [8,12,13]. For individuals with diabetes and high cardiovascular disease (CVD) risk, recommendations for blood pressure and LDL-C control are more rigorous (130/80 mmHg and 70 mg/dL, respectively).

Similar goals are recommended by the ESC/EASD Guidelines, although CVD risk is categorized into three levels of risk based on risk factors, duration of diabetes and target organ damage [14]. While making recommendations for global action, the WHO-PEN proposed essential non-communicable disease interventions for primary health care [15]. In the formation of risk factors, control is necessary for planning and tracking the quality of diabetes management in health systems, and in this regard, smoking cessation is an additional important goal. To this end, the degree of control has been reported in some nationally representative surveys [16,17], and a systematic review has summarized many similar, though frequently non-representative, reports [18].

Data about diabetes control in Brazil is currently limited, particularly when derived from population-based samples. Our aim was to estimate the prevalence of individuals with diabetes attaining the ADA recommendations for ABC control, and to investigate associated factors in a Brazilian nationwide health survey of adult residents, the National Health Survey (*Pesquisa Nacional de Saúde*—PNS) [19].

## 2. Methods

### 2.1. The PNS Survey

The 2013 PNS was a national household survey conducted by the Brazilian Institute of Geography and Statistics (IBGE) in partnership with the Ministry of Health. It used a cluster, multistage probability design with stratification of primary sampling units [20,21], these being census tracts or composition of census tracts. The target population was adult residents aged 18 years or older residing in private households throughout the country. The basic survey was divided into three parts: characteristics of the household; the household interview, answered by the household representative who provided information about all the residents of the household; and the individual interview, answered by the resident selected within the household. The latter included information on perception of health status, violence and accidents, lifestyle, and chronic diseases. Weight, height, and blood pressure were measured.

Between 2014 and 2015, biological material for laboratory determinations was obtained in return visits for a randomly selected subsample of 25% of the original primary sampling units [19]. Measurements were done at no cost to the participant by a central laboratory and included A1C and total, LDL- and HDL-Cholesterol levels.

### 2.2. Study Variables

Diabetes was defined by a positive response to the question *“Has a doctor ever diagnosed you with diabetes?”* (only outside of pregnancy). Current antidiabetic medication use was assessed by the questions *“In the last two weeks, because of diabetes, did you take oral medications to lower your sugar?”* and *“In the last two weeks, because of diabetes, did you use insulin?”*.

We estimated CVD risk using the World Health Organization cardiovascular disease risk charts [22] for the Tropical Latin America region. We considered a 10-year risk for a major CVD event of ≥20% as a high CVD risk. Individuals who responded positively to the questions: *“Has any doctor ever diagnosed you with a heart disease such as a heart attack, angina, heart failure, or other?”* or *“A doctor has given you the diagnosis of cerebral vascular accident or stroke?”* were also considered to be at high risk.

Laboratory determinations were done by a central laboratory certified by the National Glycohemoglobin Standardization Program (NGSP) [19]. A1C was performed by HPLC, and total, LDL- and HDL-Cholesterol by enzymatic, colorimetric methods. For low CVD risk individuals, we defined adequate glycemic control as an A1C < 7% and adequate blood pressure control as <140/90 mmHg. As the PNS did not collect data on statin use, we defined adequate lipid control as an LDL-C level of <100 mg/dL. For individuals deemed to be at high CVD risk, we adopted lower levels for blood pressure (<130/80 mmHg) and for LDL-C (<70 mg/dL). For smoking status, those who answered “Yes” to the question *“Do you currently smoke any tobacco products?”* were considered current smokers.

Other socio-demographic characteristics, such as sex, age, education, race, Brazilian macro-region, receipt of Brazil´s anti-poverty cash transfer, and having private health insurance, were obtained by the PNS 2013 questionnaire. Body mass index (BMI) was calculated as weight in kilograms divided by the square of height in meters.

### 2.3. Statistical Analysis

Given the response rate obtained, we used post-stratification survey weights to make results nationally representative. Following recommendations [19], these weights were calculated according to sex, age, education, and race according to region, from the total sample of the PNS. The reported proportions, means, standard deviations (SD), and confidence intervals (CI) were all weighted estimates. Association of socio-demographic and clinical characteristics with A1C levels was evaluated using a chi-square test with the Rao–Scott adjustment [23]. Mean differences between high and low CVD risk were evaluated through a *t*-test, while proportion differences were assessed by chi-square test. To evaluate factors associated with glycemic control (A1C < 7%), and with overall ABC control (attainment of two or more ABC goals), Poisson regression models were fitted to the data, with post-stratification weights. Prevalence ratios (PR) with 95% CI were estimated using a robust variance estimator, in these adjusted models. Data analysis was performed using statistical software R, version 4.0.4 [24], with the survey package [25].

## 3. Results

**Figure 1:**
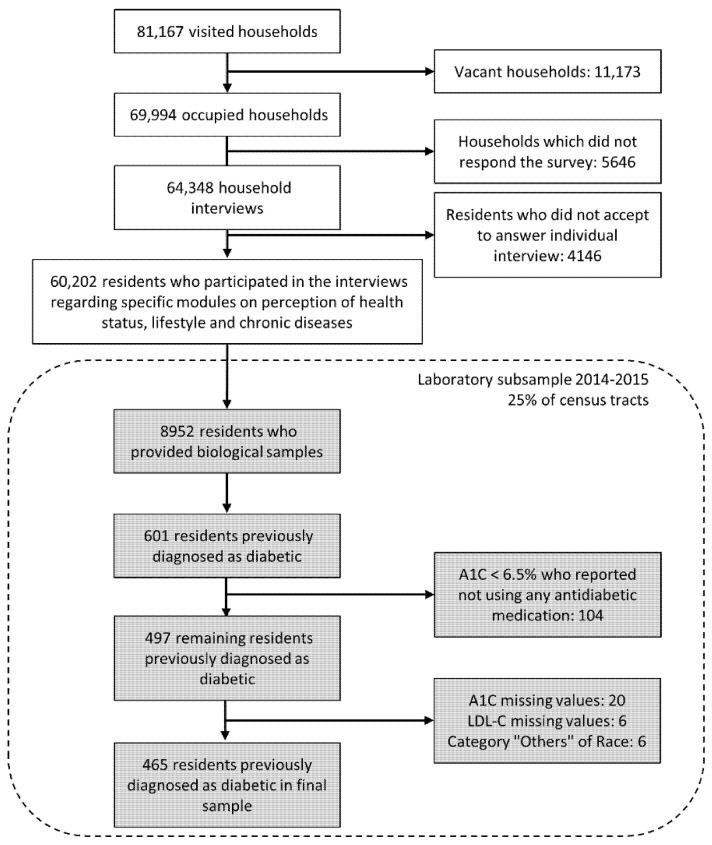
Flowchart of study participants of the laboratory subsample of the National Health Survey—Brazil, 2014–2015.

As shown in Figure 1, of the 81,167 households selected, 11,173 were vacant and 5646 did not respond, leaving 64,348 for interviews. After excluding 4146 residents who did not accept to answer the specific questionnaire or had their information rejected by the automatic coherence screening carried out by the IBGE, 60,202 (86%) were available for analyses [26]. Among the subsample selected for the collection of biological samples, 8952 (52%) provided samples [27], and among them, 601 reported having a previous diagnosis of diabetes. We excluded 104 individuals with A1C levels < 6.5% who were not using antidiabetic medication since they were unlikely to have diabetes. We further excluded those with missing A1C (*n* = 20) and LDL-C (*n* = 6) values and, due to small numbers, those declaring to be Asian or indigenous (*n* = 6). Thus, the final study sample was composed of 465 subjects with diabetes.

Mean age of participants was 43.2 years (SD = 5.2), and mean BMI was 28.9 kg/m^2^ (SD = 2.2). Among them, 22 (6.5%) reported having been diagnosed at age 30 or earlier and to be currently using insulin, thus making their diabetes most likely type 1.

Additional characteristics of the 465 participants with self-reported diabetes are shown in Table 1. Overall, participants were more frequently women, older (≥60 years), of white race/color, had completed at most elementary school, resided in Southeast Brazil, and were not a recipient of Brazil´s anti-poverty cash transfer. Approximately two-thirds had no private health insurance. Most were overweight (25–29.9 kg/m^2^) or in stage 1 obesity (30–34.9 kg/m^2^), and few smoked. Only 35 (5.8%) subjects reported having diabetes for less than one year, 38 (7.9%) were not currently taking any antidiabetic medication, and 84 (17.8%) reported a history of cardiovascular disease.

Table 1 also shows the frequency distribution of A1C values by these socio-demographic and clinical characteristics. Good glycemic control (A1C < 7%) was more common among older participants (54.9%), those living in the Center-West (50.1%) and Northeast (48.3%) macro-regions, and among those reporting the use of oral antidiabetic drugs alone (55.8%). Of note, extremely poor glycemic control (A1C ≥ 9%) was more common in the youngest subjects (32.3%), in those living in the Center-West (30.4%) and South (29%) macro-regions, in the non-obese (BMI < 30 kg/m^2^) categories, and in those reporting taking no medication for diabetes (41.3%). Additionally, those with a diabetes duration of greater than ten years tended to be more frequently (21.8%) in this category of poor control. Additionally, though not shown in the Table, 11 (11%) of those with A1C ≥ 9% were probable cases of type 1diabetes.

The upper part of Table 2 presents the mean values for the ABC risk factors, overall and by CVD risk strata. Those characterized as at high CVD risk were 84 individuals relating a history of cardiovascular disease and 13 additional subjects whose 10-year CVD risk was ≥20%. Mean A1C value was slightly lower in this high CVD risk group (7.17% vs. 7.73%). Mean blood pressure was ~5 mmHg higher and mean LDL-C approximately was 10 mg/dL lower among those with high CVD risk. The middle part of Table 2 describes categorically the distribution of blood pressure and LDL-C. Among adults with diabetes, 56.3% had blood pressure < 140/90 mmHg, and 47.0% had LDL-C < 100 mg/dL. A greater fraction of those with high CVD risk had blood pressure ≥ 160/100 mmHg (28.6% vs. 12.5%), but a lesser fraction had LDL-C ≥ 100 mg/dL (41% vs. 56.1%).

The lower part of Table 2 shows the percentage of subjects meeting target levels for each of the three risk factors and the percent not smoking. Glycemic control was more frequent among the high CVD risk group. The fact that blood pressure and LDL-C goals were more rigorous for individuals with high CVD risk contributed to the fact that the fraction of this group meeting blood pressure and cholesterol control targets was approximately half that of the remaining participants.

**Figure 2:**
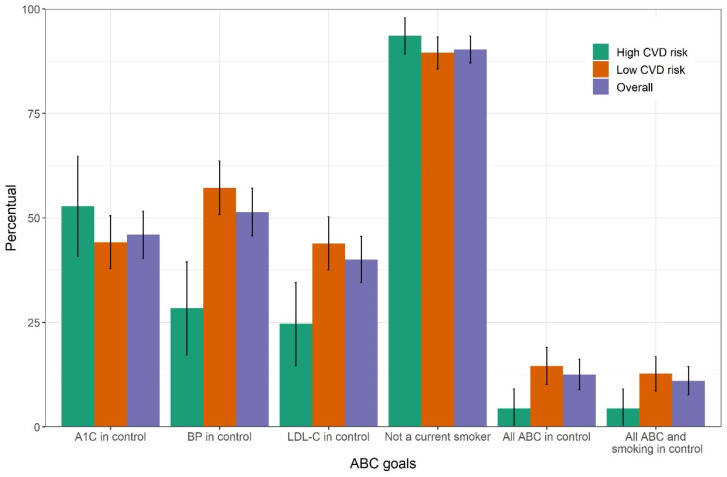
Percent attainment with 95% confidence interval for each of the three ABC goals, for the non-smoking goal and for combinations of goals among individuals with self-reported diabetes in the laboratory subsample of the Brazilian National Health Survey, 2014–2015. ABC, A1C (A), blood pressure (B) and cholesterol (C). CVD, cardiovascular disease.

Figure 2 graphically displays the level of achievement of these three individual goals as well as that of being a non-smoker (90.3%). Only 12.5% (95% CI, 8.9–16.2%) of participants achieved all three ABC goals, this fraction declining to 11% (95% CI, 7.6–14.4%) when non-smoking was also considered. Overall achievement of the combined ABC and ABC + smoking goals among those with high CVD risk was less than half that of those at lower risk, again consistent with the stricter cutoffs adopted for blood pressure and LDL-C for high CVD risk individuals.

**Figure 3:**
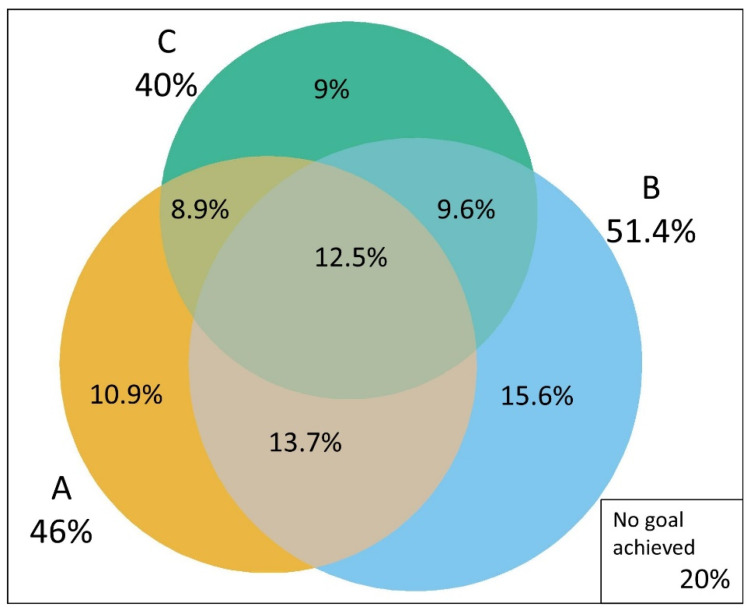
Venn diagram showing the overlap in in the attainment of treatment goals in individuals with self-reported diabetes: A1C (A), blood pressure (B) and cholesterol (C). Laboratory subsample, Brazilian National Health Survey 2014–2015.

Figure 3 shows the intersections between the sets of subjects attaining each ABC goal. In general, there was a large overlap in the individual goals achieved. Aside from the 12.5% achieving all targets, 13.7% achieved both A1C and blood pressure targets, 9.6% achieved both blood pressure and LDL-C targets, and 8.9% achieved both A1C and LDL-C targets. Only 20% of the sample did not achieve any goal.

Table 3 shows the results of Poisson analyses investigating factors associated with achieving the A1C target (left columns) and with attaining at least two of the ABC goals (right columns). Within each group, the column to the left shows associations when adjusted for age and sex, and the column to the right also adjusts for the additional variables in the table. Few associations were present. An increase of 10 years in age was associated with a 24% (Model 2; PR = 1.25; 1.14–1.35) increase in the frequency of glycemic control. Those reporting a duration of diabetes greater than 10 years had a 33% lower frequency of glycemic control (Model 2; PR = 0.67; 0.53–0.86) compared to those with a duration of 1 to 10 years. Regarding attainment of two or more ABC goals, the only associated factors were BMI and duration of diabetes. An increase of 5 kg/m^2^ on BMI was related to an 18% (Model 2; PR = 0.82; 0.73–0.92) decrease in the frequency of achieving at least two ABC goals. A time of diagnosis of 10 years or greater was associated with a 24% (Model 2; PR = 0.76; 0.59–0.98) decrease in the frequency of achieving at least two ABC goals. In these adjusted analyses, compared to those living in the Southeast Region (the richest and most populous), minimal differences in the prevalence of good control were seen, statistically significantly so only for reaching at least two goals for those living in the Center-West region. None of the other characteristics shown in Table 1 were associated with either A1C or ABC control.

**Figure 4:**
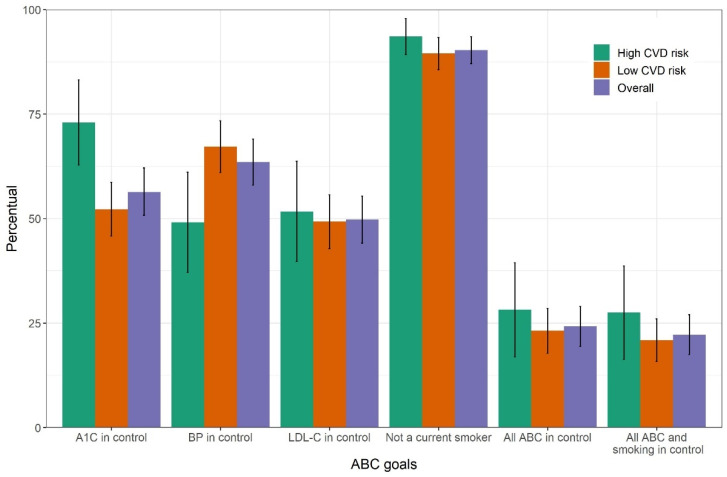
Sensitivity analysis. Percent attainment with 95% confidence interval for each of the three ABC goals, for the non-smoking goal and for combinations of goals among individuals with self-reported diabetes in the laboratory subsample of the Brazilian National Health Survey, 2014–2015. Goals here were relaxed for those aged 60 or over. ABC, A1C (A), blood pressure (B) and cholesterol (C). CVD, cardiovascular disease.

As the established goals may be difficult and sometimes unrealistic to be achieved by certain groups of patients, we conducted a sensitivity analysis considering the following more relaxed goals for individuals aged 60 years and over: A1C < 8%, blood pressure < 150/90 mmHg, and LDL-C < 110 mg/dL. The percentage of individuals achieving the three goals improved slightly, with 56% (95% CI, 50.8–62.1%) achieving glycemic control, 63% (95% CI, 58–69%) blood pressure control goal, and 50% (95% CI, 44.1–55.4%) LDL-C control goal. The fraction of individuals achieving all three ABC goals doubled to 24% (95%, 19.4–29.0%) (Figure 4).

## 4. Discussion

In this sample of Brazilian adults with known diabetes, after excluding the 17% with normal A1C values off medication, approximately half reached each of the three ABC goals. However, only one in ten achieved all three goals. Among those with high CVD risk, control of A1C was more frequent, and that of blood pressure and LDL-C, for which stricter goals are recommended, less so. More frequent A1C control was associated with older age and a shorter duration of diabetes. Achievement of at least two ABC goals was associated with lower BMI and a shorter duration of diabetes. No clear clustering of specific risk factor targets achieved was present when control of more than one factor was achieved. Non-smoking was achieved by more than 90%.

This is the first report of risk factors control in a nationally representative sample of Brazilians with known diabetes. The fact that 90.3% of the sample reached a non-smoking goal, which was higher than the percentage seen in other surveys [16,28], is unlikely to be primarily due to clinical interventions. Rather, this finding reflects the dramatic declines in smoking since 1989 in Brazil following the implementation of multiple public policies against tobacco, including a ban on tobacco advertising, legislation on tobacco-free environments, increased taxes and taxation, and increased warnings [29]. 

The fraction of individuals reaching glycemic control (46%; A1C < 7%) was similar to that seen in the 2015–2018 US NHANES [16] (50.5%) and in the Korean NHANES [17] (52.6%). However, the fraction of Brazilians with a controlled blood pressure was lower (51.4%) than that found in the US NHANES (70.4%; <140/90 mmHg) [16] and the Korean NHANES (68.4%; <140/85 mmHg) [17]. For the LDL-C goal, the fraction in control (40%) was similar to that in the Korean NHANES (44.2%; LDL-C < 100 mg/dL) [17], but lower than that in the US NHANES (55.7%; non-HDL-C < 130 mg/dL) [16]. The prevalence of control in our sample was also lower than that reported in a Spanish nationwide survey [30], which found that 70.9% of those with known diabetes had an A1C < 7%, a 60.0% blood pressure < 140/90 mmHg and 35.6% an LDL-C < 100 mg/dL.

Attainment of all ABC goals was generally poor in all studies, particularly when considering stricter thresholds: among Brazilians, 12.5% and 24% achieved all three goals when considering ADA and more relaxed goals, respectively. In the US NHANES 22.2% of individuals simultaneously achieved all three targets (A1C < 7%, blood pressure < 140/90 mmHg, and non–high-density lipoprotein cholesterol <130 mg/dL) [16]. In the Korean NHANES, with a more stringent target for glycemic control (A1C < 6.5%), and with targets of blood pressure <140/85 mmHg, and LDL-C below 100 mg/dL, 8.4% of subjects reached good control of all three targets [17].

In a meta-analysis of 24 studies of ABC control with data from 20 countries [18] (many not representative of the general population living with diabetes, which complicates their generalization), target achievement was 42.8% for glycemic control (vs. the 46% here reported), 29.0% for blood pressure (vs. 51.4%), and 49.2% for LDL-C (vs. 40%). Of note, few of these studies were done outside of North America and Europe. In a non-representative study in Brazil [31], glycemic control (24%) was less frequent. In a report investigating type 2 diabetes in Latin America, glycemic control was similar (43.5%) to that described here [32]. 

Within this scenario of few with diabetes achieving multiple risk factor targets in Brazil and elsewhere, the benefits attainable with their greater control merits reflection. First, with respect to smoking, which is a major risk factor for cardiovascular disease in diabetes [33], effective treatments exist [34], and non-smoking should be stimulated to all and prescribed when patients demonstrate interest.

Second, regarding the risk associated with the degree of glucose control, a metanalysis of observational studies showed a nadir in mortality at an A1C of 6–7%, with those within 7–8%, 8–9%, and above 9% showing greater mortality risks of 17%, 31% and 69%, respectively [35]. Thus, while these data justify aiming for an A1C below 7%, they also illustrate that larger benefit from improved glycemic control can be expected in those with increasingly high A1C values, especially in those over 8%.

Third, regarding the benefits of multifactorial interventions to simultaneously achieve ABC goals, the landmark 2008 report of the Steno-2 randomized trial found a 59% decrease in cardiovascular events and a 46% decrease in overall deaths [6]. However, a recent meta-analysis of 19 trials did not support an important benefit of intensified multifactorial therapy, showing no reduction in all-cause mortality (RR = 1) [11], with a 10% relative reduction in non-fatal myocardial infarction being the only clear benefit found. Similarly, a 10-year follow-up of the effect of intensification of treatment in a large, randomized trial of screen-detected cases of diabetes showed only non-significant reductions of 13% in first cardiovascular events and 10% in mortality. These studies do not support the amplification of benefits that simultaneous intensification of therapies targeting multiple risk factors might have been expected to produce.

Thus, while our findings unquestionably alert to the necessity to improve ABC goals achievement in Brazil, the absence of an amplified benefit of multifactorial intervention permits more flexibility to personalize targets within a more patient-centered approach. Cogent arguments can be made to relax goals in certain groups—particularly those with diabetes onsetting at older ages as well as in other settings of decreased life expectancy.

More relaxed control of hyperglycemia is supported by a meta-analysis of several large clinical trials [36], showing that the benefit of tighter glucose control over 5 years was close to nil and was overshadowed by an increased risk of hypoglycemia. While based on studies of more than a decade ago, the medications used—including metformin, sulfonylureas, and insulin—are still the mainstay of glucose management in Brazil´s national health system as well as in the care of diabetes in most settings around the world. With respect to hypertension, the benefit of lowering blood pressure beyond 140/90 mmHg in those with diabetes initially at levels above 140/90 mmHg has yet to be demonstrated [9]. With respect to hypercholesterolemia, lipid lowering therapy has been shown to be of major benefit to high risk individuals, including those with diabetes [37]. However, a large fraction of those with diabetes, as demonstrated by our data, do not have high (≥20%) 10-year cardiovascular risk. For such low-risk individuals, the literature shows that many, perhaps as many as 250, would need treatment with a statin to prevent one death [38].

In contrast, much can be gained by improving risk factor levels of those who are in the poorest control/greatest risk. The greatest gains in blood pressure control were achieved when blood pressure levels were highest [39]. Evidence-based guidelines of international authorities prioritize the identification and treatment of high overall cardiovascular risk [15,40]. As shown here, an appreciable fraction of Brazilian adults with diabetes are in extremely poor control: 20.1% had an A1C > 9%, 15.8% blood pressure > 160/100 mmHg and 6.8% with LDL-C > 160 mg/dL, and 21% are at high CVD risk.

We found few characteristics that identified those not reaching good control: for hyperglycemia, being younger and having a greater duration of diabetes, consistent with previous findings [31]; for ABC goals, having a greater BMI and a longer duration of diabetes, consistent with previously reported findings [28]. The finding of BMI influencing risk is not surprising, especially as central obesity has long been known to predict greater CVD risk [41]. Though limited sample size decreased our ability to detect relevant correlates of control, the absence of associations with race, educational level, and private health insurance highlights the positive role of Brazil´s national health system and its broad primary care network in the management of diabetes.

In sum, within the context of gains from control reported in the literature, the more marginal gains attainable from treatment to target must be evaluated together with individual patient priorities, and focus would be better placed on those with risk factors clearly out of control [42] or at high risk, within a personalized approach in the management of risk factors [13]. Advancements have been made in this regard, particularly for goal setting in [12,14,15]. Policy making must also consider their local priorities and include population strategies to confront non-communicable diseases [43].

An additional finding meriting discussion is that 17% of our original sample had an A1C below the threshold for diabetes, despite the absence of pharmacological treatment, and were therefore excluded from analyses. This considerable frequency alerts the necessity to question the diagnosis of diabetes in such cases, since regression from diabetes is common [44], especially when initial ascertainment was not confirmed. Many, as appears the case in our sample, may believe that they have diabetes based on past testing when in fact, they do not.

Our study has limitations. First, the small number of individuals diagnosed with diabetes in the PNS 2014–2015 laboratory subsample hinders our ability to analyze population correlates of control, especially with respect to a disparity in control across racial subgroups and geographical macro-regions. Second, diabetes was defined by self-report, making misclassification possible. However, our exclusion of those with A1C < 6.5% who were not using diabetes medication should minimize this risk. Third, due mainly to the difficulty of locating participants in return visits for blood sample collection and the refusal at times of the selected resident to collect biological material, the large percentage of losses to our sample may have biased results. However, the post-stratification weights we applied to maintain sample representativeness hopefully reduced the magnitude of this problem. Additionally, the PNS did not collect data on statin use, which makes it difficult to compare our findings with current ADA recommendations for lipid control. With respect to our definition of CVD risk, other criteria to diagnose high risk in diabetes would result in some shift in the percent meeting the ABC goals. As high risk individuals must meet more rigorous ABC control cutoffs, definitions of risk that define more participants as at high risk will result in a lower fraction reaching the combined ABC goal. Finally, comparisons of the level of control across studies are frequently only approximate, given the different cutoffs applied.

Despite these limitations, our study has important strengths. It permits a comprehensive evaluation of the status of ABC risk factor and smoking control in Brazilian adults with diabetes, considering both rigid and more flexible goals. Our report is based on a nationally representative sample, thus avoiding biases related to the degree of referral typical of studies based on those attending specific health centers. Our findings provide a surveillance baseline for these levels of control. They help fill the gap of low- and middle-income countries in which representative studies of control have been conducted and can serve for comparison of future studies of other similarly situated countries.

## 5. Conclusions

Achievement of ABC treatment goals in this population-based sample of subjects with known diabetes was poor, with only half of subjects achieving each target and few all of them. This poor achievement and the appreciable fraction of the sample with notably poor control, in consonance with that seen in other countries, indicates the need to improve access and quality of treatment. Lack of support in the literature for added benefit from efforts aimed at simultaneous control of multiple risk factors suggests that treatment of these risk factors in diabetes should be patient-centered, prioritizing goal achievement within the context of individual patient desires, risks, and realities.

## Figures and Tables

**Table 1:** Socio-demographics and clinical characteristics of individuals with self-reported diabetes overall and according to glycated hemoglobin (A1C) level: Laboratory subsample, National Health Survey—Brazil, 2014–2015.

Characteristic		A1C (%)				*p* Value ^†^
	Overall	<7	7 to <8	8 to <9	≥9	
Overall, *n* (%)	465 (100)	225 (46.0)	73 (19.8)	60 (14.1)	107 (20.1)	
Sex, *n* (%)						0.12
Female	308 (61.5)	158 (49.9)	40 (15.9)	37 (12.8)	73 (21.4)	
Male	157 (38.5)	67 (39.6)	33 (26.0)	23 (16.3)	34 (18.1)	
Age group (years), *n* (%)						0.005
18–44	53 (11.5)	25 (25.3)	6 (26.7)	4 (15.7)	18 (32.3)	
45–59	168 (38.1)	70 (40.4)	24 (16.4)	18 (13.7)	56 (29.5)	
60+	244 (50.4)	130 (54.9)	43 (20.8)	38 (14.1)	33 (10.2)	
Race, *n* (%)						0.31
White	191 (52.1)	96 (48.3)	36 (22.4)	23 (12.5)	36 (16.9)	
Black or Brown	274 (47.9)	129 (43.5)	37 (17.0)	37 (15.9)	71 (23.6)	
Education, *n* (%)						0.97
Elementary or less	345 (70.3)	158 (45.3)	56 (19.7)	49 (14.1)	82 (20.9)	
Above elementary school	120 (29.7)	67 (47.5)	17 (19.9)	11 (14.3)	25 (18.3)	
Region, *n* (%)						0.03
North	64 (3.5)	30 (46.1)	8 (12.7)	9 (15.3)	17 (25.9)	
Northeast	145 (20.1)	70 (48.3)	23 (15.8)	16 (11.2)	36 (24.7)	
Southeast	125 (55.0)	61 (45.4)	28 (25.1)	18 (15.1)	18 (14.4)	
South	59 (13.0)	26 (42.0)	7 (11.7)	10 (17.2)	16 (29.0)	
Center-West	72 (8.4)	38 (50.1)	7 (9.9)	7 (9.6)	20 (30.4)	
Receiving cash transfer, *n* (%)						0.84
No	431 (94.2)	208 (46.4)	71 (20.0)	57 (14.0)	95 (19.6)	
Yes	34 (5.8)	17 (39.3)	2 (16.2)	3 (17.1)	12 (27.4)	
Private health insurance, *n* (%)						0.21
No	329 (68.4)	148 (45.7)	51 (17.0)	43 (14.5)	87 (22.8)	
Yes	136 (31.6)	77 (46.6)	22 (25.8)	17 (13.3)	20 (14.3)	
Body mass index group (kg/m^2^), *n* (%)						<0.001
<25	98 (21.3)	55 (50.9)	6 (6.0)	17 (22.4)	20 (20.8)	
25–29.9	168 (34.6)	75 (44.0)	25 (17.8)	21 (12.9)	47 (25.3)	
30–34.9	127 (29.2)	67 (52.8)	23 (20.7)	13 (10.8)	24 (15.7)	
≥35	72 (14.9)	28 (30.0)	19 (42.5)	9 (11.7)	16 (15.8)	
Current smoker, *n* (%)						0.33
No	416 (90.3)	205 (45.9)	61 (19.3)	56 (15.1)	94 (19.7)	
Yes	49 (9.7)	20 (46.5)	12 (24.4)	4 (4.9)	13 (24.2)	
Years since diabetes diagnosis, *n* (%)						0.13
0–1	35 (5.8)	20 (59.4)	7 (21.5)	1 (1.2)	7 (18.0)	
1–10	217 (46.0)	117 (50.5)	34 (20.0)	22 (10.9)	44 (18.6)	
10+	213 (48.3)	88 (40.0)	32 (19.4)	37 (18.8)	56 (21.8)	
Current antidiabetic medication, *n* (%)						<0.001
None	38 (7.9)	10 (20.8)	8 (24.7)	6 (13.2)	14 (41.3)	
Oral antidiabetic drugs alone	341 (71.2)	193 (55.8)	45 (17.7)	41 (12.0)	62 (14.5)	
Insulin alone	19 (6.0)	8 (38.1)	3 (12.9)	2 (25.4)	6 (23.6)	
Both insulin and oral antidiabetic drugs	67 (14.9)	14 (15.7)	17 (30.0)	11 (20.2)	25 (34.1)	
History of cardiovascular disease, *n* (%)						0.21
No	381 (82.2)	181 (44.6)	59 (18.8)	48 (14.5)	93 (22.1)	
Yes	84 (17.8)	44 (52.3)	14 (24.4)	12 (12.4)	14 (11.0)	

With the exception of the column “Overall”, percentages are with respect to row totals. ^†^ *p* values refer to the association of socio-demographic and clinical characteristics with A1C levels.

**Table 2:** Levels of glycated hemoglobin (A1C), blood pressure (BP), and low-density lipoprotein-cholesterol (LDL-C), distribution of blood pressure and LDL-C categories, and percent reaching the target value (“in control”) of each among individuals with self-reported diabetes: Laboratory subsample, National Health Survey—Brazil, 2014–2015.

Characteristic	Overall	High CVD Risk	Low CVD Risk	*p* Value ^†^
	(*n* = 465)	(*n* = 97)	(*n* = 368)	
	Mean (SD)	Mean (SD)	Mean (SD)	
A1C (%)	7.62 (1.86)	7.17 (1.76)	7.73 (1.87)	0.01
Systolic BP (mmHg)	136.8 (21.2)	141.0 (27.4)	135.8 (19.2)	0.13
Diastolic BP (mmHg)	80.4 (11.8)	78.6 (12.7)	80.8 (11.5)	0.23
LDL-C (mg/dL)	104.2 (34.3)	95.7 (33.6)	106.4 (34.2)	0.02
BP (mmHg)	*n* (%)	*n* (%)	*n* (%)	0.01
<130/80	153 (30.4)	24 (28.4)	129 (31.0)	
130/80 to <140/90	120 (25.9)	25 (24.4)	95 (26.3)	
140/90 to <160/100	119 (27.9)	19 (18.7)	100 (30.2)	
≥160/100	73 (15.8)	29 (28.6)	44 (12.5)	
LDL-C (mg/dL)	*n* (%)	*n* (%)	*n* (%)	0.15
<100	214 (47.0)	56 (59.0)	158 (43.9)	
100–129	151 (33.3)	25 (28.2)	126 (34.6)	
130–159	70 (12.8)	12 (7.1)	58 (14.3)	
≥160	30 (6.8)	4 (5.6)	26 (7.1)	
Attained control	*n* (%; 95%CI)	*n* (%; 95%CI)	*n* (%; 95%CI)	
A1C	225 (46.0; 40.3–51.6)	49 (52.8; 40.8–64.8)	176 (44.2; 37.9–50.5)	0.22
BP	248 (51.4; 45.7–57.1)	24 (28.4; 17.2–39.5)	224 (57.2; 50.8–63.6)	<0.001
LDL-C	183 (40.0; 34.5–45.6)	25 (24.7; 14.7–34.6)	158 (43.9; 37.6–50.3)	0.004
Non-smoking	416 (90.3; 87.1–93.5)	87 (93.6; 89.3–97.9)	329 (89.5; 85.6–93.3)	0.19

^†^*p* values refer to the differences between high and low CVD risk groups.

**Table 3:** Factors associated with glycemic control (glycated hemoglobin < 7%) and attainment of glycemic, blood pressure and cholesterol (ABC) control (attainment of two or more goals) among those with self-reported diabetes: Laboratory subsample, National Health Survey—Brazil, 2014–2015.

Characteristic	A1C < 7%		Achieving ≥ 2 Goals	
	Model 1	Model 2	Model 1	Model 2
	PR (95% CI)	PR (95% CI)	PR (95% CI)	PR (95% CI)
Sex (reference: Female)				
Male	0.84 (0.64–1.08)	0.79 (0.61–1.01)	0.87 (0.66–1.14)	0.79 (0.60–1.03)
Age (increase of 10 years)	**1.18 (1.08–1.29)**	**1.24 (1.14–1.35)**	1.03 (0.94–1.14)	1.05 (0.95–1.15)
Race (reference: White)				
Black or Brown	0.97 (0.77–1.23)	0.94 (0.75–1.19)	0.81 (0.63–1.05)	0.80 (0.62–1.02)
Education (reference: Elementary or less)				
Above elementary school	1.23 (0.95–1.58)	1.24 (0.97–1.59)	1.27 (0.97–1.67)	1.29 (0.98–1.68)
Region (reference: Southeast)				
North	1.12 (0.79–1.59)	1.12 (0.78–1.62)	0.97 (0.68–1.38)	1.08 (0.75–1.56)
Northeast	1.04 (0.81–1.34)	1.04 (0.80–1.35)	0.83 (0.62–1.10)	0.84 (0.62–1.12)
South	1.01 (0.71–1.44)	0.97 (0.68–1.39)	0.96 (0.66–1.39)	0.90 (0.62–1.32)
Center-West	1.18 (0.88–1.59)	1.15 (0.86–1.55)	1.28 (0.95–1.72)	**1.37 (1.03–1.83)**
Receiving cash transfer (reference: No)				
Yes	1.01 (0.56–1.84)	0.94 (0.52–1.70)	0.94 (0.53–1.66)	0.81 (0.46–1.42)
Private health insurance (reference: No)				
Yes	0.99 (0.77–1.27)	0.93 (0.72–1.21)	1.24 (0.96–1.60)	1.15 (0.88–1.51)
Body mass index (increase of 5 kg/m^2^)	0.93 (0.83–1.03)	0.91 (0.82–1.02)	**0.85 (0.76–0.95)**	**0.82 (0.73–0.92)**
Current smoker (reference: No)				
Yes	0.88 (0.60–1.31)	0.92 (0.64–1.33)	0.83 (0.57–1.21)	0.90 (0.64–1.27)
Years since diabetes diagnosis (reference: 1–10)				
0–1	1.22 (0.86–1.73)	1.20 (0.84–1.72)	0.69 (0.39–1.20)	0.68 (0.41–1.12)
10+	**0.68 (0.53–0.88)**	**0.67 (0.53–0.86)**	0.79 (0.60–1.04)	**0.76 (0.59–0.98)**
History of cardiovascular disease (reference: No)				
Yes	1.05 (0.79–1.40)	1.09 (0.83–1.43)	0.76 (0.53–1.10)	0.79 (0.55–1.13)

Model 1: adjusting for age and sex; Model 2: adjusting for age, sex, race, education, body mass index and years since diabetes diagnosis. Note: results in bold are statistically significant at the 5% level.

## Data Availability

Publicly available datasets were analyzed in this study. These data can be found here: https://www.pns.icict.fiocruz.br/bases-de-dados/ (accessed on 21 July 2021).
